# A Treatise on the Chronic Inflammations and Displacements of the Unimpregnated Uterus

**Published:** 1871-04

**Authors:** 


A Treatise on the Chronic Inflammations and Displacements of the Unimpregnated Uterus. By Wm. H. Byford, A.M., M.D. Second edition, revised and enlarged. Philadelphia: Lindsay & Blakiston, Publishers. S. C. Griggs & Co., Chicago.

This little work comprises fifteen chapters, devoted to the consideration of the following subjects: Sympathetic accompaniments of uterine disease, local symptoms, etiology, prognosis, complications of inflammation of cirvex, position of inflammation, progress and terminations, diagnosis, general treatment, local treatment, nitrate of silver and its substitutes, treatment of sub-mucous inflammation, displacements—their philosophy and treatment.

Reports of several interesting cases are given in an appendix. The first edition of the work received the commendation of the medical press, and its rapid sale is sufficient evidence of the approval of the profession generally. The present edition is much improved in matter and style.

